# Passive Q-Switching by Cr^4+^:YAG Saturable Absorber of Buried Depressed-Cladding Waveguides Obtained in Nd-Doped Media by Femtosecond Laser Beam Writing

**DOI:** 10.3390/ma11091689

**Published:** 2018-09-12

**Authors:** Gabriela Croitoru (Salamu), Nicolaie Pavel

**Affiliations:** Laboratory of Solid-State Quantum Electronics, National Institute for Laser, Plasma and Radiation Physics, 077125 Magurele, Ilfov, Romania; gabriela.croitoru@inflpr.ro

**Keywords:** lasers, solid-state, neodymium, Q-switched, waveguides, channeled, micro-optical devices

## Abstract

We report on laser performances obtained in Q-switch mode operation from buried depressed-cladding waveguides of circular shape (100 μm diameter) that were inscribed in Nd:YAG and Nd:YVO_4_ media by direct writing with a femtosecond laser beam. The Q-switch operation was realized with a Cr^4+^:YAG saturable absorber, aiming to obtain laser pulses of moderate (few μJ) energy at high (tens to hundreds kHz) repetition rate. An average power of 0.52 W at 1.06 μm consisting of a train of pulses of 7.79 μJ energy at 67 kHz repetition rate, was obtained from a waveguide realized in a 4.8 mm long, 1.1-at % Nd:YAG ceramics; the pulse peak power reached 1.95 kW. A similar waveguide that was inscribed in a 3.4 mm long, 1.0-at % Nd:YVO_4_ crystal yielded laser pulses with 9.4 μJ energy at 83 kHz repetition rate (at 0.77 W average power) and 1.36 kW peak power. The laser performances obtained in continuous-wave operation were discussed for each waveguide used in the experiments. Thus, a continuous-wave output power of 1.45 W was obtained from the circular buried depressed-cladding waveguide inscribed in the 1.1-at %, 4.8 mm long Nd:YAG; the overall optical-to-optical efficiency, with respect to the absorbed pump power, was 0.21. The waveguide inscribed in the 1.0-at %, 3.4 mm long Nd:YVO_4_ crystal yielded 1.85 W power at 0.26 overall optical efficiency. This work shows the possibility to build compact laser systems with average-to-high peak power pulses based on waveguides realized by a femtosecond (fs) laser beam direct writing technique and that are pumped by a fiber-coupled diode laser.

## 1. Introduction

The experiments performed by Davis et al. [1] demonstrated that a femtosecond (fs) laser beam can induce stable damages and changes of the refractive index in various glasses. The written tracks presented an increased index of refraction in comparison with that of the bulk medium, which allowed light propagation within the track itself; such an optical device is of interest for telecommunications, to build planar as well as three-dimensional photonic devices [2]. On the other hand, in the case of crystals the stress field induces an increase of the refractive index in the zones adjacent to that where laser irradiation is performed [3]. Based on this finding, wave-guiding was realized between two such written tracks that were positioned at a narrow distance (up to a few tens of μm) from each other; single-transverse mode operation was obtained in this way [4,5].

Power-scaling of a waveguide realized by an fs-laser beam can be achieved using a different approach, that is, the buried depressed-cladding waveguide. A tubular waveguide is obtained with this technique, by using a core of the crystal with unmodified properties around which many parallel tracks are written [6]. Efficient laser emission at several wavelengths was reported from various buried depressed-cladding waveguides that were inscribed in different laser crystals, such as Nd:YAG and Nd:YVO_4_ (with emission at 1.06 μm), Tm-doped glass or Tm:YAG ceramics (with emission from 1.9 μm near-infrared up to 3.4 μm mid-infrared), Pr:YLF and Pr:SrAl_12_O_19_ (with emission directly into the visible spectrum), in Nd- or Yb-doped tungstate crystals or in ZnSe and ZnS crystals. Detailed reviews of the works and achievements in the field of ultra-fast laser inscribing were done by Chen and Vázquez de Aldana [7], Choudhury et al. [8], and by Meany et al. [9]. Recently, we used the pump with fiber-coupled diode laser to achieve watt-level emission in continuous-wave (cw) operation from buried depressed-cladding waveguides that were inscribed by an fs-laser beam in Nd:YAG and Nd:YVO_4_ [10,11]. Among the many recent results we can mention the laser emission in the 2.0 μm range in waveguides written in Ho:YAG [12], the interest for emission into the visible spectrum from depressed-cladding waveguides inscribed in the Pr:YLF crystal [13], or the realization of a hexagonal optical-lattice-like cladding structure in the Tm:KLu(WO_4_)_2_ crystal with efficient laser emission around 1.8 μm [14].

Most of the waveguides mentioned before were operated in free-generation regime (using cw or quasi-cw pumping). On the other hand, the possibility to obtain pulses with high-peak power is also of high interest; for example, such a device could be integrated in various miniature systems, for efficient non-linear conversion, or it could be of interest in some medical applications. Consequently, Q-switch operation with different media having saturable absorption (SA) at the laser wavelength of interest has been studied in such waveguides realized by fs-laser beam writing. Okhrimchuck [15] was the first to report Q-switch operation of a circular, buried depressed-cladding waveguide that was realized in a diffusion-bonded Nd:YAG/Cr^4+^:YAG composite crystal. Laser pulses with 10 μJ energy and 1-ns duration at 1-kHz repetition rate (10-mW average power) were obtained from a circular waveguide with 110 μm diameter using the pump with a fiber-coupled diode laser. Cr^4+^:YAG SA was also used to obtain passive Q-switching from two-wall type waveguides that were inscribed in Nd:YAG/Cr^4+^:YAG or Yb:YAG/Cr^4+^:YAG composite crystals [16,17]. The average power at 1.06 μm reached 300 mW (at 1 μJ laser pulse energy), being obtained from a waveguide realized in Nd:YAG/Cr^4+^:YAG, with 25 μm separation between the walls and that was pumped with a Ti:sapphire laser [16]. A power of 0.61 W at 1.03 μm, with laser pulses of 2.7 μJ energy and 3.4 ns duration, was yielded by a two-wall waveguide that was written in Yb:YAG/Cr^4+^:YAG [17].

Besides the use of a composite structure for the laser medium and the SA component, Q-switch operation was also obtained from discrete laser elements, in which the SA was positioned close to the laser medium. Thus, a semiconductor saturable absorber mirror was employed to achieve Q-switch laser emission from a circular, depressed-double-cladding waveguide that was inscribed in Nd:YAG ceramic [18], as well as from a two-track waveguide written in Yb:YAG crystal [19]. Graphene was considered for the Q-switch in such waveguides also. Thus, a multi-line type waveguide inscribed in Nd:YVO_4_ was Q-switched with graphene SA coated on a quartz plate [20]. A monolayer of graphene was coated for Q-switch operation on the exit surface of a two-track Yb:YAG waveguide [21] and a circular depressed-cladding waveguide inscribed in Ho:YAG was operated at 2.1 μm in Q-switch mode-locking regime with a graphene based saturable output coupler [22]. Among other recent results we can mention the following: carbon nanotubes were used to obtain Q-switched laser emission from a channel waveguide inscribed in Yb:YAG [23]; a circular depressed-cladding waveguide inscribed in Nd:YAG was operated in Q-switch regime by MoS_2_ SA [24], as well as with molybdenum diselenide and tungsten diselenide membranes covered on silica wafers [25]; vanadium dioxide was investigated for Q-switch operation in depressed-cladding waveguides inscribed in Nd:YVO_4_ [26]. The use of such SA materials [23,24,25] resulted, however, in Q-switch laser operation with low pulse energy, of a few tens up to about one hundred nJ and long duration, from tens of ns up to several hundred ns.

Recently we reported high-average power Q-switch laser operation from circular, depressed-cladding waveguides that were inscribed in Nd:YAG/Cr^4+^:YAG composite crystals; worthy of mention was that the optical pump was made with a fiber-coupled diode laser [27]. An average power of 1.1 W at 1.06 μm was obtained from a 150-μm diameter waveguide that was inscribed in a 1.0-at % Nd:YAG/Cr^4+^:YAG (Cr^4+^:YAG SA with initial transmission T_i_ = 0.70); the pulse energy and duration was 15.7 μJ and 3.9 ns, respectively, corresponding to a peak power of 4 kW. The use of a composite Nd:YAG/Cr^4+^:YAG medium can lead to a compact device, but it can also be an expensive choice. In addition, the use of discrete elements has the advantage of obtaining laser pulses with different characteristics by replacing only the Cr^4+^:YAG SA crystal, and not all of the system. In the present work we extend the previous investigations to Q-switch operation by discrete Cr^4+^:YAG SA of depressed-cladding waveguides that were inscribed in Nd:YAG ceramics and Nd:YVO_4_ crystals. Average output power P_ave_ = 0.52 W at 1.06 μm with laser pulses of energy E_p_ ~ 7.8 μJ and peak power P_p_ = 1.95 kW at 67-kHz repetition rate was obtained from a 100 μm diameter waveguide inscribed in a 1.1-at % Nd:YAG ceramics; the Cr^4+^:YAG SA initial transmission was T_i_ = 0.89. A similar waveguide realized in a 1.0-at % Nd:YVO_4_ crystal yielded pulses with energy E_p_ = 9.4 μJ and peak power P_p_ = 1.36 kW; the average power was P_ave_ = 0.77 W. Characteristics of the laser pulses obtained from waveguides inscribed in other Nd:YAG and Nd:YVO_4_ active media and employing Cr^4+^:YAG SA with different T_i_ are given.

## 2. Waveguides Description

In the present experiments we used some of the waveguides realized and employed in our previous works [10,11]. For a better understanding, we remind the reader that the waveguides were inscribed with a Clark-MRX-2101 chirped-pulsed amplified system that delivers laser pulses at 775 nm with 200 fs pulse duration at 2 kHz repetition rate and up to 0.6 mJ energy. The fs-laser pulse energy suitable for writing each waveguide was chosen by an optical configuration comprising a half-wave plate, a polarizer, and several calibrated neutral filters. Typically, the beam was focused in the laser medium at a spot size of a few-μm diameter. The writing process was monitored with a camera.

**Figure 1:**
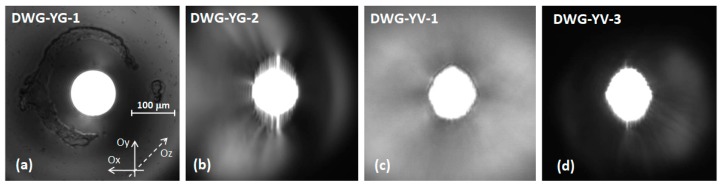
Photos of circular (100 μm diameter) buried depressed-cladding waveguides inscribed in 1.1-at % Nd:YAG ceramics: (**a**) DWG-YG-1 (4.5 mm long); (**b**) DWG-YG-2 (length of 4.8 mm) and in Nd:YVO_4_ crystals; (**c**) DWG-YV-1 (0.5-at % Nd:YVO_4_, 6.9 mm long); (**d**) DWG-YV-3 (1.0-at % Nd:YVO_4_, 3.4 mm long).

Figure 1 presents images of the exit surfaces of some waveguides while the pump radiation was coupled in each waveguide on the opposite side. Two waveguides, each with diameter of 100 μm, were inscribed in Nd:YAG ceramics (Baikowski Co., Ltd., Chiba, Japan). The first one was obtained in a 1.1-at % Nd:YAG ceramics of 4.5 mm length using a helical-moving technique developed in our group [28]; this waveguide, shown in Figure 1a, is denoted by DWG-YG-1. A second waveguide was written in a 4.8 mm long, 1.1-at % Nd:YAG ceramics by the classical writing technique developed by Okhrimchuck [6,15]. The waveguide consisted of an unmodified core of Nd:YAG around which many tracks were realized, at a distance of ~5 μm between two consecutive tracks; this waveguide will be called DWG-YG-2 (Figure 1b). An evaluation of the change of the refractive index induced in Nd:YAG by the fs-laser beam was made following the method described in Ref. [4]. Thus, a HeNe laser beam was coupled in each waveguide and the maximum incident angle at which no change of the transmitted power was occurring was determined. Following this technique of calculus it was concluded that the approximate change in the Nd:YAG refractive index Δn, between the unchanged refractive index of waveguide core and the average refractive index of the waveguide wall, was around 1 × 10^−3^.

Three other waveguides, all having a diameter of 100 μm, were obtained by the classical writing technique in a-cut Nd:YVO_4_ crystal (FOKtec Photonics, Inc., Fuzhou, China): DWG-YV-1 in a 6.9 mm long, 0.5-at % Nd:YVO_4_ crystal (Figure 1c); DWG-YV-2 in a 4.6 mm long, 0.7-at % Nd:YVO_4_ crystal and DWG-YV-3 in a 3.4 mm long, 1.0-at % Nd:YVO_4_ crystal (Figure 1d). The surfaces of each medium were polished after the writing process and then coated as antireflection (reflectivity R < 0.25%) at the lasing wavelength (λ_em_) of 1.06 μm and with high transmission (transmission T > 0.99) at the pump wavelength (λ_p_) of ~0.81 μm.

## 3. Results

### Laser Emission Experiments

In the laser experiments we used a plane–plane resonator. The resonator rear mirror (HRM) was coated for high reflectivity (reflectivity, R > 0.998) at λ_em_ and for high transmission (transmission, T > 0.98) at λ_p_; this mirror was placed on a translation stage that has enabled its positioning very close to one surface of each waveguide. The pump was made through the HRM with a fiber-coupled diode laser (LIMO Co., Dortmund, Germany) that was operated in cw mode. The fiber end (100-μm diameter, numerical aperture NA = 0.22) was imaged into each waveguide with a collimating achromatic lens with focal distance of 50 mm and a focusing achromatic lens with 30 mm focal distance. The coupling efficiency of the pump beam into the waveguide was evaluated as a unit. Each laser medium was wrapped in indium foil and embedded in a copper holder; the holder temperature was kept at 20 °C by using a Peltier element cooled with re-circulated water. Uncoated Cr^4+^:YAG SA crystals (Cryslaser, Inc., Chengdu, China) with initial transmission T_i_ = 0.89, T_i_ = 0.84, and T_i_ = 0.79 were tested in the Q-switch experiments. Each Cr^4+^:YAG was placed (with positioning systems) as close as possible to the waveguide surface opposite to the one through which the pump was made, and was followed by the resonator out-coupling mirror (OCM). Several OCMs with transmission T between 0.01 and 0.10 were used for determining the optimum emission characteristics.

**Figure 2:**
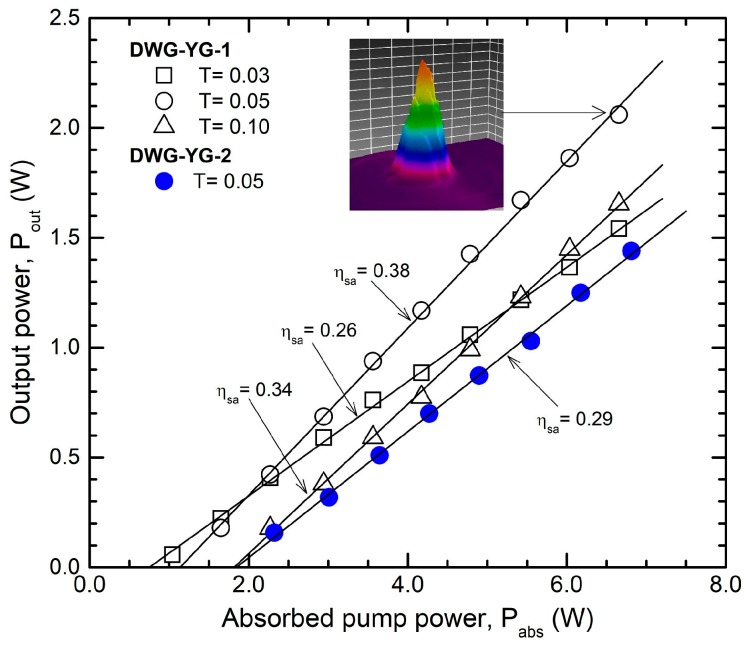
Cw output power, P_out_ versus absorbed pump power, P_abs_ for the waveguides inscribed in Nd:YAG. T: Out-coupling mirror (OCM) transmission. Inset is a 3D distribution of the laser beam at the maximum P_out_.

The laser emission performances measured in cw regime from the waveguides inscribed in Nd:YAG are shown in Figure 2 (in these experiments the Cr^4+^:YAG SA was removed from the resonator). With an OCM of T = 0.05 the waveguide DWG-YG-1 yielded maximum output power P_out_ = 2.05 W for an absorbed pump power P_abs_ = 6.65 W; this corresponds to an optical-to-optical efficiency, η_oa_ (with respect to P_abs_) of ~0.31. The slope efficiency (with respect to P_abs_) was η_sa_ = 0.38. In the case of waveguide DWG-YG-2 the output power was limited to P_out_ = 1.45 W for P_abs_ = 6.80 W (i.e., η_oa_ = 0.21); the laser operated with slope η_sa_ = 0.29 (OCM with T = 0.05). The differences in output performances between DWG-YG-1 (the waveguide written by helical moving techniques) and DWG-YG-2 (the waveguide inscribed by classical step-by-step method) can be explained by lower losses in the case of the first waveguide. Thus, based on a Findlay-Clay analysis [29] of cw emission characteristics it was concluded that the resonator round-trip loss was L_i_ = 0.03 for waveguide DWG-YG-1, but much higher, L_i_ = 0.10, for waveguide DWG-YG-2. We comment that in the case of laser emission in bulk Nd:YAG loss L_i_ was evaluated, for both Nd:YAG media, to be lower than 0.01. On the other hand, losses L_i_ for waveguides are in agreement with propagation losses determined at 632.8 nm, these being of 0.6 dB/cm for DWG-YG-1 and ~1.5 dB/cm for DWG-YG-2 [10]. In our previous investigations it was showed that laser emission from such waveguides has a high M^2^ factor, indicating multimode transverse operation [28,30,31]. The inset of Figure 2 presents the 3D shape of the laser beam at the maximum output power (P_out_ = 2.05 W, the waveguide DWG-YG-1), suggesting similar multimode distribution of the laser beam.

**Figure 3:**
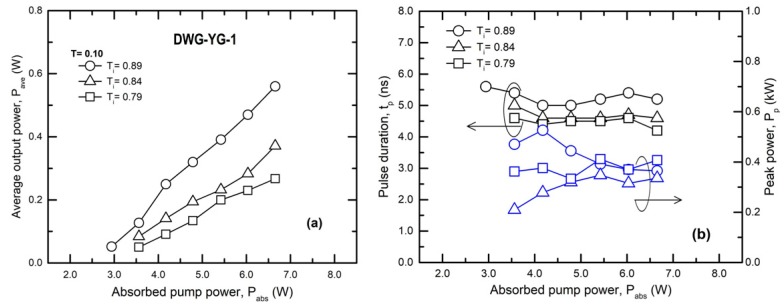
Q-switch operation for waveguide DWG-YG-1: (**a**) Average output power, P_ave_; (**b**) Pulse duration, t_p_ and laser pulse peak power, P_p_. T_i_: Initial transmission of Cr^4+^:YAG SA.

For waveguide DWG-YG-1 the Q-switch regime was investigated with Cr^4+^:YAG of initial transmission T_i_ = 0.89, 0.84, and 0.79 and with an OCM of transmission T = 0.10 (for which the highest average output power was obtained). The results are shown in Figure 3. For the Cr^4+^:YAG with T_i_ = 0.89 the maximum average output power was P_ave_ = 0.56 W (Figure 3a). At this level the laser ran at a repetition rate r_p_ = 295 kHz, from which the laser pulse energy was evaluated as E_p_ = 1.89 μJ. A fast UPD-35-IR2-D photodiode (Alphalas, Göttingen, Germany) with a short (<35 ps) rise time and a Tektronix DPO7254 digital oscilloscope (2.5 GHz bandwidth, 40 GS/s sample ra1te) were used to measure the laser pulse duration, t_p_. The pulse duration was t_p_ = 5.2 ns and therefore pulse peak power was calculated as P_p_ = 0.36 kW (Figure 3b). In the case of Cr^4+^:YAG with T_i_ = 0.84 the average power was limited to P_ave_ = 0.37 W; the pulse energy was E_p_ = 1.55 μJ, and pulse duration narrowed to t_p_ = 4.6 ns (i.e., P_p_ ~ 0.34 kW). A slightly shorter pulse, t_p_ = 4.2 ns, was measured for the Cr^4+^:YAG SA with T_i_= 0.79; the power decreased at P_ave_ = 0.27 W, the laser ran at r_p_ = 156 kHz with energy E_p_ = 1.71 μJ (or peak power P_p_ ~ 0.41 kW). It is noticeable that the duration of the laser pulse and its energy were nearly constant throughout the entire pump range (Figure 3b), in accordance with theory for such a passive Q-switch laser. Unfortunately damage of the waveguide coating was observed (this can be seen in Figure 1a); therefore further experiments were performed only for the Cr^4+^:YAG SA with T_i_ = 0.89.

**Figure 4:**
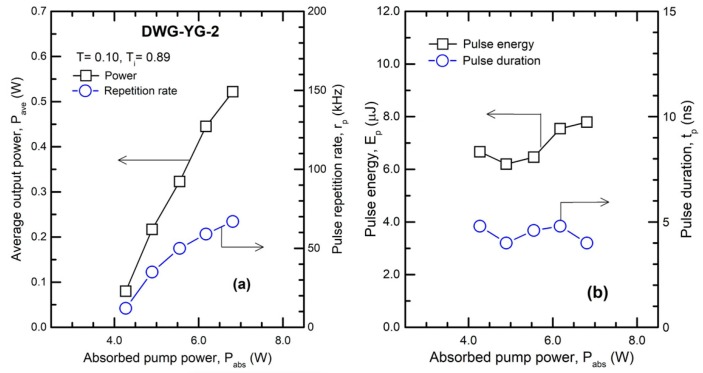
Q-switch operation for waveguide DWG-YG-2 and Cr^4+^:YAG with T_i_ = 0.89: (**a**) Average power, P_ave_ and pulse repetition rate, r_p_; (**b**) laser pulse energy, E_p_ and duration, t_p_.

The main results obtained with waveguide DWG-YG-2 and the Cr^4+^:YAG of T_i_ = 0.89 are given in Figure 4. One could see that due to high losses (L_i_ ~ 0.08) lasing was obtained only from a quite high pump power at threshold, of about 4 W. The laser yielded P_ave_ = 0.52 W at a repetition rate r_p_ = 67 kHz (Figure 4a); thus, at this point the laser pulse energy was calculated as E_p_ = 7.79 μJ. The pulse duration was t_p_ = 4 ns (Figure 4b) giving a pulse peak power P_p_ = 1.95 kW. There is a wider variation of laser pulse parameters with the pump power (Figure 4b), most likely due to the thermal effects induced by the pump in the laser medium and the variation with them of the waveguide properties.

**Figure 5:**
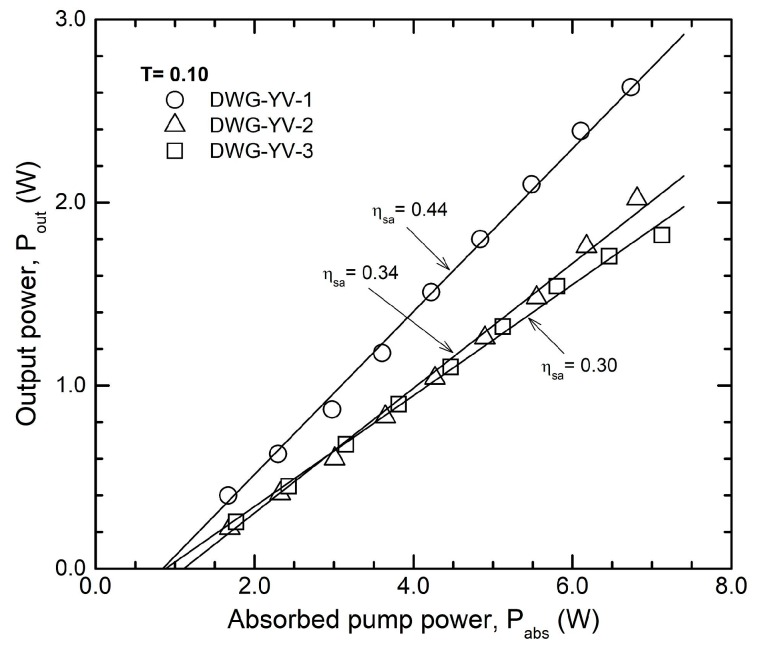
Cw operation for the waveguides inscribed in Nd:YVO_4_, OCM with T = 0.10.

The characteristics of cw laser emission yielded by the waveguides inscribed in Nd:YVO_4_ crystals are given in Figure 5; the best performances were obtained with an OCM of transmission T = 0.10. The waveguide DWG-YV-1 delivered maximum power at 1.06 μm, P_out_ = 2.90 W for P_abs_ = 6.75 W (i.e., η_oa_ ~ 0.43); a good slope efficiency η_sa_ = 0.44 was determined.

The results obtained in cw regime with all the waveguides investigated in this work are summarized in Table 1. It can be seen that power P_out_ = 2.0 W and P_out_ = 1.85 W were measured from waveguide DWG-YV-2 and DWG-YV-3, respectively; the corresponding slope efficiency η_sa_ was 0.34 and 0.30. As in the case of Nd:YAG, a Findlay-Clay analysis concluded that the resonator round-trip loss was L_i_ ~ 0.06 for the waveguide DWG-YV-1 and nearly the same, L_i_ ~ 0.10, for the other two waveguides inscribed in Nd:YVO4. The laser beam at the maximum output power was polarized E||c axis (π polarization) with extinction ratio better than 100:1, similar to the results reported in Ref. [11].

**Figure 6:**
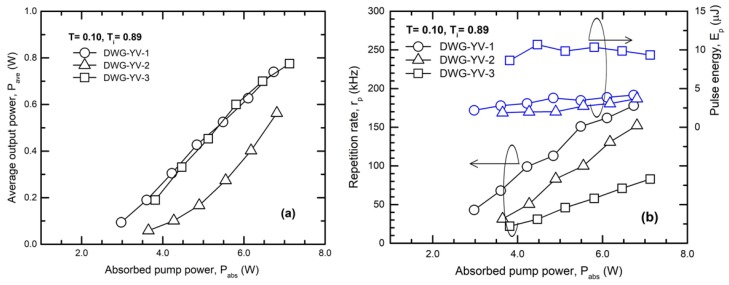
Q-switch operation for the waveguides inscribed in Nd:YVO_4_: (**a**) Average power, P_ave_; (**b**) laser pulse repetition rate, r_p_ and pulse energy, E_p_. OCM with T = 0.10 and Cr^4+^:YAG with T_i_ = 0.89.

The average power, P_ave_ outputted in Q-switch regime (Cr^4+^:YAG SA with T_i_ = 0.89) by the waveguides inscribed in Nd:YVO_4_ and the resonator with an OCM of T= 0.10 is plotted in Figure 6a versus P_abs_. The highest power P_ave_ = 0.77 W was delivered by waveguide DWG-YV-3 at a repetition rate r_p_ = 83 kHz (Figure 6b). The pulse energy reached E_p_ = 9.4 μJ and the pulse peak power was calculated as P_p_ = 1.36 kW (the pulse duration was t_p_ = 6.8 ns). Finally, the characteristics of the Q-switch laser pulses obtained in this work with the Cr^4+^:YAG SA of T_i_ = 0.89 are summarized in Table 2. One can see that the waveguide DWG-YV-1 yielded laser pulses with E_p_ = 4.15 μJ at high repetition rate, r_p_ = 178 kHz and quite low peak power P_p_ = 0.29 kW (pulse duration, t_p_ = 14 ns). The use of a shorter DWG-YV-2 waveguide decreased the pulse duration at t_p_ = 8.2 ns whereas the repetition rate was r_p_ = 152 kHz; the laser pulse energy and peak power was E_p_ = 3.68 μJ and 0.45 kW, respectively.

## 4. Discussion

The spectroscopic properties of interest for the Q-switch regime differ for Nd:YAG and Nd:YVO_4_. Thus, for Nd:YAG the emission cross-section, σ_g_ in the 1 μm range is moderate, σ_g_ = 2.6 × 10^−19^ cm^2^ and the luminescence lifetime of the ^4^F_3/2_ emitting level at 1.0-at % Nd-doping level is long, τ_f_ ~ 225 μs [32]. In case of Nd:YVO_4_ the emission-cross section is much larger, σ_g_ = 14.1 × 10^−19^ cm^2^ (in πpolarization), whereas the lifetime is shorter, τ_f_ ~ 84 μs for 1.0-at % Nd:YVO_4_. Thus, due to their spectroscopic characteristics, Nd:YAG can provide Q-switch laser pulses of high energy at tens to hundreds of kHz repetition rate, while Nd:YVO_4_ is expected to yield pulses with lower energy, but at much higher repetition rate.

Several experiments performed in all bulk Nd:YAG ceramics and Nd:YVO_4_ crystals confirmed these statements. For example, the Q-switch laser built with the 4.5 mm long, 1.1-at % Nd:YAG, the Cr^4+^:YAG SA with T_i_ = 0.89, and an OCM with T = 0.05 delivered P_ave_ = 2.3 W for the pump P_abs_ = 6.65 W; the laser ran at r_p_ = 312 kHz, indicating a pulse energy E_p_ ~ 7.4 μJ. Furthermore, average power P_ave_ = 1.6 W (at P_abs_ = 6.75 W) was obtained from the 6.9 mm long, 0.5-at % Nd:YVO_4_ crystal, the Cr^4+^:YAG SA of T_i_ = 0.89 and an OCM of T = 0.10. The repetition rate was r_p_ = 1720 kHz and therefore the laser pulse energy amounted to E_p_ = 0.93 μJ. These results could be easily modeled by the theory of the passive Q-switch regime [33,34]. However, the waveguides used in the experiments, both those inscribed in Nd:YAG or in Nd:YVO_4_, yielded laser pulses with comparable (μJ level) energy. Therefore, further analysis is necessary in order to understand this behavior. The influence of thermal effects on the waveguide dimensions; an accurate determination of each waveguide and resonator losses; the impact of the pump radiation that is not absorbed in the waveguide on the properties of the Cr^4+^:YAG SA medium [35], or the variation with temperature of Nd:YAG and Nd:YVO_4_ emission cross sections [36,37], which was shown to have a great impact on the emission performances of such lasers passively Q-switched by the Cr^4+^:YAG SA [38,39], could be considered in modeling.

## 5. Conclusions

In this work we reported on the Q-switch laser pulse characteristics obtained from circular (100 μm diameter) buried depressed-cladding waveguides that were inscribed in Nd:YAG ceramics and Nd:YVO_4_ single crystals by direct writing with an fs-laser beam. Compared to a previous paper [27], in which Nd:YAG and Cr^4+^:YAG SA media were bonded in a composite structure, in the present investigations we considered discrete elements in order to obtain a greater variety of laser pulses, mainly by changing the Cr^4+^:YAG SA. For the pump level used in the experiments (close to or slightly below P_abs_ = 7 W), the average power at 1.06 μm reached P_ave_ = 0.52 W from a waveguide inscribed in a 1.1-at % Nd:YAG ceramic of 4.8 mm length. This device, which was built with a Cr^4+^:YAG of T_i_ = 0.89 and a resonator OCM of T = 0.05, delivered pulses with energy E_p_ = 7.79 μJ and short 4.0 ns duration; the pulse peak power was P_p_ = 1.95 kW. Laser pulses with close peak power, P_p_ ~ 1.36 kW at 83 kHz repetition rate were yielded by a waveguide that was written in a 3.4-mm long, 1.0-at % Nd:YVO_4_ crystal, and that was placed in a resonator with the Cr^4+^:YAG of T_i_ = 0.89 and an OCM with T = 0.10; the pulse energy was high, E_p_ = 9.4 μJ. Worthy of mention is that the pump was made with a fiber-coupled diode laser; this proves the potential to build compact laser systems with average-to-high peak power pulses based on waveguides realized by fs-laser beam direct writing technique. Further work could consider modeling of such passively Q-switch Nd:YAG-Cr^4+^:YAG systems.

## Figures and Tables

**Table 1:** Characteristics of cw laser emission at 1.06 μm yielded by the circular, buried depressed-cladding waveguides realized in Nd:YAG (OCM with T = 0.05) and in Nd:YVO_4_ (OCM with T = 0.10).

Laser Medium	Waveguide	Cw Output Power, P_cw_ (W)	Absorbed Pump Power, P_abs_ (W)	Absorption Efficiency, η_a_	Optical Efficiency, η_oa_	Slope, η_sa_
1.1-at % Nd:YAG, 4.5 mm	DWG-YG-1	2.05	6.65	0.85	0.31	0.38
1.1-at % Nd:YAG, 4.8 mm	DWG-YG-2	1.45	6.80	0.87	0.21	0.29
0.5-at % Nd:YVO_4_, 6.9 mm	DWG-YV-1	2.90	6.75	0.86	0.43	0.44
0.7-at % Nd:YVO_4_, 4.6 mm	DWG-YV-2	2.0	6.80	0.87	0.29	0.34
1.0-at % Nd:YVO_4_, 3.4 mm	DWG-YV-3	1.85	7.10	0.91	0.26	0.30

**Table 2:** Q-switch laser emission at 1.06 μm, OCM with T = 0.10, Cr^4+^:YAG with T_i_ = 0.89.

Laser Medium	Waveguide	Average Output Power, P_ave_ (W)	Repetition Rate, r_p_ (kHz)	Pulse Energy, E_p_ (μJ)	Pulse Duration, t_p_ (ns)	Peak Power, P_p_ (kW)
1.1-at % Nd:YAG, 4.5 mm	DWG-YG-1	0.56	295	1.89	5.2	0.36
1.1-at % Nd:YAG, 4.8 mm	DWG-YG-2	0.52	67	7.79	4.0	1.95
0.5-at % Nd:YVO_4_, 6.9 mm	DWG-YV-1	0.74	178	4.15	14.0	0.29
0.7-at % Nd:YVO_4_, 4.6 mm	DWG-YV-2	0.56	152	3.68	8.2	0.45
1.0-at % Nd:YVO_4_, 3.4 mm	DWG-YV-3	0.77	83	9.4	6.8	1.36
